# Bacteriospermia and its association with seminal fluid parameters and infertility in infertile men, Kerman, Iran: A cross-sectional study

**DOI:** 10.18502/ijrm.v20i3.10712

**Published:** 2022-04-21

**Authors:** Parastou Heidari Pebdeni, Fereshteh Saffari, Toraj Reza Mirshekari, Sareh Ashourzadeh, Moslem Taheri Soodejani, Roya Ahmadrajabi

**Affiliations:** ^1^Department of Medical Microbiology (Bacteriology & Virology), Afzalipour Faculty of Medicine, Kerman University of Medical Sciences, Kerman, Iran.; ^2^Medical Mycology and Bacteriology Research Center, Kerman University of Medical Sciences, Kerman, Iran.; ^3^Afzalipour Clinical Center for Infertility, Kerman University of Medical Sciences, Kerman, Iran.; ^4^Center for Healthcare Data Modeling, Departments of Biostatistics and Epidemiology, School of Public Health, Shahid Sadoughi University of Medical Sciences, Yazd, Iran.

**Keywords:** Chlamydia trachomatis, Infertility, Mycoplasma, Semen analysis, Ureaplasma.

## Abstract

**Background:**

The role of genital *Ureaplasma* species, genital *Mycoplasma* *(M)* species, and *Chlamydia (C.) trachomatis,* the most prevalent sexually transmitted bacteria, in male infertility are still not clear. Different reports about the impact of these bacteria on semen quality are controversial.

**Objective:**

This study was proposed to determine the frequency of bacteriospermia in men and investigate the relationship between the presence of these bacteria and semen quality using molecular assay.

**Materials and Methods:**

In this cross-sectional study, 200 semen samples obtained from men attending the research and clinical centers for fertility in Kerman, Iran, between July and December 2019 were analyzed for semen volume, progressive motility, non-progressive motility, total progressive motility, and viability according to the World Health Organization guidelines. The polymerase chain reaction was used for the detection of related bacteria.

**Results:**

The mean values of volume, progressive motility, non-progressive motility, total progressive motility, and viability were significantly lower in infertile men (p < 0.001). Statistically significant correlations were observed between the presence of *M. genitalium* and progressive sperm motility, *M. hominis* and semen volume, *Ureaplasma parvum* and the sperm normal form, and *C. trachomatis* and the sperm progressive motility and viability. Logistic regression analysis showed that *M. genitalium *(OR = 8.06, p < 0.001) and *C. trachomatis *(OR = 16, p = 0.01) were significantly associated with male infertility.

**Conclusion:**

During the infertility assessment, clinicians should consider of role *C. trachomatis* and *M. genitalium* in male infertility. Screening test particularly for asymptomatic individuals is recommended.

## 1. Introduction

Sexually transmitted infections are of significant concern to investigators and clinicians of reproductive medicine (1). Even though the influence of certain genital bacterial infections on sperm function and whole spermatogenesis have been proposed, the role of these infections as the significant causes of male infertility is still controversial (2). The most prevalent sexually transmitted bacteria are *Chlamydia trachomatis *(*C*.* trachomatis*), *Ureaplasma urealyticum *(*U. urealyticum*), *U. parvum*, *Mycoplasma hominis* (*M. hominis*), and *M. genitalium*.

The prevalence of genital chlamydial infections has been estimated to be 50-70%. However, most infections are asymptomatic, and are neither diagnosed nor treated (2, 3). The impact of *Chlamydia* on semen parameters and its role in male infertility has been investigated in different studies (4, 5). However, no definite conclusion has been acquired. One study has shown that *C. trachomatis* infection is not associated with semen abnormalities, while another study has shown that contamination with *C. trachomatis* is associated with decreased sperm concentrations (6, 7).

Although genital mycoplasmas and ureaplasmas are natural residents of the male urethra, these microorganisms, particularly *U. urealyticum*, can contaminate seminal fluid during ejaculation and contribute to both genital infections and male infertility (8). However, the exact role of these agents in male infertility remains unknown (2). The study of Rybar and colleagues displayed that *Mycoplasma* has the highest adverse effect on sperm quality, such as concentration, motility, and morphology (7). In contrast, in another study, genital *Ureaplasma* or *Mycoplasma* were not significantly related to semen abnormalities (8).

Determination of the possible relationship between infertility and the presence of *C. trachomatis* and genital ureaplasmas/mycoplasmas in the seminal fluid may have valuable consequences in the public health of different geographical areas (8). Due to the social and psychological effects and high costs of infertility treatments, accurate diagnosis and timely infection treatment are important to prevent future complications. So, screening strategies seem to be necessary.

To the best of our knowledge, no adequate research has been done for the frequencies of *C. trachomatis*, *Ureaplasma species* (spp.) and *Mycoplasma *spp. infections in infertile men in Kerman, Iran. This research was proposed to determine the frequency of the above sexually transmitted bacteria in semen samples and investigate the relationship between the presence of these bacteria and semen quality using molecular assay.

## 2. Methods and Materials 

### Subjects

From July to December 2019, a total of 100 infertile men attending the research and clinical centers for fertility in Kerman (southeast of Iran) were included in this cross-sectional study. “Infertility was defined as a failure to conceive after at least 12 months of unprotected sexual intercourse” (9). Inclusion criteria for male infertility were as follows: infertility without female factor subfertility, lack of hormonal abnormalities, and reproductive system abnormalities (varicocele, hydrocele, undescended testis, or inguinal hernia), the absence of clinical signs of genitourinary tract infections, and history of infertility and abnormal semen parameter. None of the participants had consumed any antibiotics for the last two wk (2, 9). As the control group, semen specimens from 100 fertile men with normal semen parameters whose wives had non-assisted pregnancies in the past (2) were obtained from the same clinical centers during the same period.

For sample size estimating, the statistical formula was conducted in which p, z ${}_{1-}$ β, and z ${}_{1-\alpha }$ were 0.05, 0.84, and 1.96 respectively, and the sample size was determined to be about 100 (cases = 100, controls = 100).

### Collection of semen specimens

Seminal fluids were collected either by self or assisted masturbation after 2-7 days of sexual abstinence. Before collecting the samples, participants were recommended to wash their hands and genital area with water and soap. Samples were collected in sterile plastic containers and transported to the microbiology laboratory within one hr. Following this, they were placed in the incubator for 15-30 min for liquefaction. They then were subjected to routine semen analysis and polymerase chain reaction (PCR) to detect mentioned bacteria.

### Semen analysis

Semen analysis was performed according to World Health Organization guidelines to investigate the following parameters: appearance, semen volume, morphology, round cell, progressive motility, liquefaction, sperm concentration, and vitality.

### DNA extraction and PCR

Bacterial DNA was extracted using an appropriate nucleic acid extraction kit (AmpliSensⓇ RIBO-prep, Moscow, Russia) according to the manufacturer's protocol for the isolation of bacterial DNA from clinical materials. The absorption of the extracted DNA was measured at 260 nm and 280 nm using NanoDrop NDe1000 Spectrophotometer (NanoDrop Technologies, Wilmington, DE, USA) to confirm the quality of the product. The extracted DNA was first examined for the human β-globin gene to check that there were no PCR inhibitors in the samples.

BgloF (5 ' - CAACTTCATCCACGTTCACC-3 ' ) and BgloR (5 ' GAAGAGCCAAGGACAGGTAC-3 ' ) were used to amplify a 268-bp fragment in the exon 1 of the human β-globin (2). All β-globin-positive samples were examined for the presence of *Ureaplasma* spp., *U*. *urealyticum*, *U. parvum*, and *M. hominis* by conventional PCR (2, 10, 11) and semi-nested PCR for *M. genitalium* (12) and using an appropriate detection kit for *C. trachomatis*.

### Detection of *M. hominis* and genital ureaplasmas by PCR

Amplification of *ure A-B*, multiple banded antigens (*mba*), and *16SrRNA* by PCR assay were performed to identify *Ureaplasma *spp.,* U*. *urealyticum* or *U. parvum*, and *M. hominis*, respectively. A total volume of 25-μl PCR mixture including 2 μl of bacterial DNA, 0.5 μl (5 pM) of each oligodeoxynucleotide primer, 12.5 μl of 2 × Master Mix Red (Ampliqon, Odensem, Denmark) was used for PCR. The primer quest software tool (http://www.ncbi.nlm.nih.gov/Gene) was used to check the specificity of primers shown in table I. After performing PCR according to the programs shown in table I, the PCR products were electrophoresed.

### Detection of *M. genitalium*

The first conventional PCR was carried out for amplification of a 286-bp adhesion DNA fragment with the F1 and R1 primers and temperature program described in table I. Amplification of a 194-bp adhesion DNA fragment with F1 and R2 primers (Table I) was carried out by using semi-nested-PCR as described previously (12). Amplification was conducted with an initial denaturation at 95 C for five min, followed by 40 cycles at 94 C for 30 sec, 54 C for 45 sec, 72 C for 60 sec, and a final extension at 72 C for five min.

### Detection of *C. trachomatis*

According to the manufacturer's instructions*, C. trachomatis* was detected using an appropriate detection kit (Iranian Gene Fanavar Institute, Tehran, Iran).

### Sequencing 

The Sanger dideoxy chain termination technique on an Applied Biosystems 3730/3730Xl DNA Analyzer (Applied Biosystems, Foster City, CA, USA) by Bioneer Company was used for sequencing the PCR products. The primers used for sequencing were the same as PCR, except for C*. trachomatis* (Table I). Homology of the resultant sequences was checked using the NCBI-NIH BLAST search program.

**Table 1:** PCR primers and cycling parameters for detection of sexually transmitted bacteria


**Bacterium**	**Gene target**	**Primer/sequences (5 ${}^{ʹ}$ - 3 ${}^{ʹ}$ )**	**PCR condition**	**PCR product (bp)**	**Reference**
Ureaplasma **spp** ***.***	*Ure A-B*	F: ACG ACG TCC ATA AGC AAC T R: CAA TCT GCT CGT GAA GTA TTA C	30 sec 94ºC, 30 sec 60ºC, 1 min 72ºC	425	2
Ureaplasma urealyticum	*mba*	F: TTT GCA AAA CTA TAA ATA GAC AC R: TTT GTT GTT GCG TTT TCT G	1 min 94ºC, 1 min 52ºC, 1 min 72ºC	363	10
Ureaplasma parvum	*mba*	F: AAT AAA TCT TAG TGT TCA TAT TTT TTT AC R: GTA AGT GCA GCA TTA AAT TCA ATG	1 min 94ºC, 1 min 52ºC, 1 min 72ºC	327	10
Mycoplasma hominis	*16SrRNA*	F: CAA TGG CTA ATG CCG GAT ACG C R: GGT ACC GTC AGT CTG CAA T	35 sec 95ºC, 35 sec 55ºC, 30 sec 72ºC	334	11
Mycoplasma genitalium	*adhesion*	F1: AGT TGA TGA AAC CTT AAC CCC TTG G R1: CCG TTG AGG GGT TTT CCA TTT TTG C R2: GAC CAT CAA GGT ATT TCT CAA CAG C	1 min 94ºC, 30 sec 64ºC, 1 min 72ºC	194	12
PCR: Polymerase chain reaction, bp: Base pair					

### Ethical considerations

The present study was approved by the Ethics Committee of the Research Council of Kerman University of Medical Sciences, Kerman, Iran (Code: IR.KMU.REC.1398.208). Written informed consent was obtained from all the participants.

### Statistical analysis

Data analysis was done using IBM SPSS Statistics version 16. Descriptive statistics were done by calculating mean, standard deviation, and frequency. Fisher's exact test was used to assess the relationship between the prevalence of some bacteria and infertility situations. The logistic regression was conducted to predict the impact of bacterial infections on male infertility. The significance level of 5% was considered in all statistical analyses.

## 3. Results

A total of 200 infertile and fertile men participated in this study. The mean age of infertile men was 35.33 yr (range 21-60; SD ± 7.38), while the mean age of fertile men was 34.29 yr (range 17-60, SD ± 7.3). Although the mean age of infertile men was 1.04 yr higher than those in the fertile group, this difference was not significant (p = 0.73).

Sixty one percent of specimens (122 of 200) were positive for one or more studied bacteria. Overall 74% of total men carried *Ureaplasma *spp., of which 5% carried *U. urealyticum *(serovars 2, 10, 11, 12), 40% carried *U. parvum *(serovars 1, 3, 14), and the remained ones carried the other serovars that were not detectable by the used primers (Table II). Simultaneous infection with *Ureaplasma *spp*. *and *M. genitalium* was observed in 7% of infertile men (Table III).

There was a statistically significant difference only in the presence of *M. genitalium* and *C. trachomatis* in infertile men vs. fertile ones (Table II).

**Figure 1:**
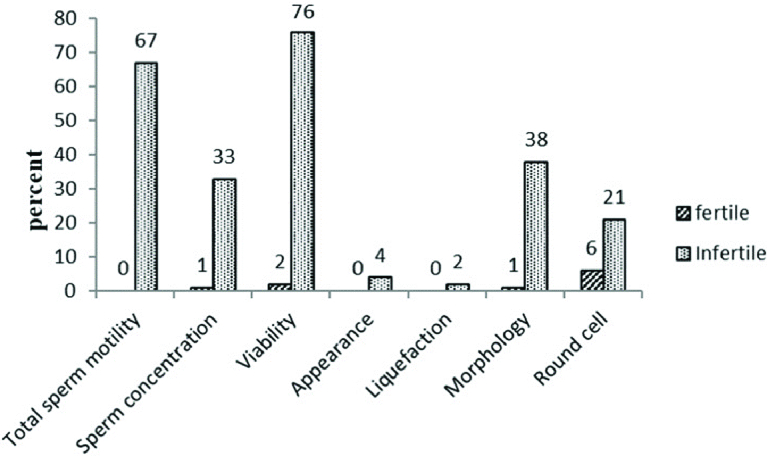
The comparison of abnormal semen characteristics in the fertile and infertile men.

Semen characteristics in all participants showed that 98% of them had normal semen appearance (gray or opalescent), and in 99.5% of the men, semen was liquefied within less than 60 min. Sperm morphology, concentration, viability, total sperm motility and the percent of round cells were also in the normal range in 80.5%, 83.0%, 61.0%, 66.5%, and 86.5% of specimens, respectively. Figure 1 shows the frequency of abnormal semen characteristics separately.

The semen variables in the fertile and infertile men are summarized in table IV. The mean values of volume, progressive motility, non-progressive motility, sperm concentration, total progressive motility, and viability were significantly lower in infertile men than fertile men.

We studied the effects of the presence of *M. genitalium, M. hominis, Ureaplasma *spp*., U. urealyticum, U. parvum, *and* C. trachomatis* on semen quality in infected vs. uninfected infertile men. A statistically significant correlation was observed between infection with *M. genitalium* and the percentage of progressive sperm motility (22% in infected infertile men vs. 33% in uninfected infertile men, p = 0.04), infection with *M. hominis* and semen volume (2.8 mL in infected infertile men vs. 3.27 mL in uninfected infertile men, p = 0.03) and infection with *U. parvum* and the normal form of sperm (67.5% in infected infertile men vs. 84.7% in uninfected infertile men, p = 0.02). Additionally, the correlation between infection with *C. trachomatis* and the progressive motility of sperm (15.67% in infected infertile men vs. 32.27% in uninfected infertile men, p = 0.03) and also the percentage of live sperm per milliliter was significant (22% in infected infertile men vs. 63.5% in uninfected infertile men, p = 0.03).

Logistic regression analysis (multivariate analysis) showed that *M. genitalium* and *C. trachomatis* were significantly associated with male infertility, so that the risk for infertility in infected men with *M. genitalium* was 8.06 times higher than in non-infected ones, and in infected men with *C. trachomatis*, it was 16 times higher than in non-infected individuals (OR = 16, CI: 1.67-153179) (Table V).

**Table 2:** Association between the presence of tested bacteria in semen specimens


**Bacteria**	**Fertile**	**Infertile**	**P-value**
Mycoplasma genitalium	2 (12.5)	14 (87.5)	< 0.001
Mycoplasma hominis	16 (69.6)	7 (30.4)	0.07
Ureaplasma **spp.**	33 (44.6)	41 (55.4)	0.31
Ureaplasma urealyticum	3 (60.0)	2 (40.0)	0.99
Ureaplasma parvum	16 (40.0)	24 (60.0)	0.22
Chlamydia trachomatis	0 (0)	9 (100)	< 0.001
Data presented as n (%). Fisher's exact test, P < 0.05 was considered statistically significant			

**Table 3:** Frequency of co-existence of tested bacteria in semen specimens


**Bacteria**	**Groups**	
	**Fertile**	**Infertile**
Ureaplasma **spp.** + M. hominis	7 (7%)	3 (3%)
U. urealyticum + *M. hominis*	1 (1%)	1 (1%)
U. parvum + *M. hominis*	4 (4%)	1 (1%)
U. parvum + *U. urealyticum*	1 (1%)	0
M. hominis + *M. genitalium*	0	1 (1%)
Ureaplasma **spp.** + M. genitalium	0	7 (7%)
U. parvum + *M. genitalium*	0	3 (3%)
C. trachomatis + *M. genitalium*	0	1 (1%)
C. trachomatis + *M. hominis*	0	2 (2%)
U. parvum + *C. trachomatis*	0	3 (3%)
Ureaplasma **spp.** + C. trachomatis	0	5 (5%)
Ureaplasma **spp.** + M. genitalium + M. hominis	0	1 (1%)
M. hominis + *M. genitalium* + *U. parvum*	0	1 (1%)
C. trachomatis + *Ureaplasma* **spp.** + *M. hominis*	0	1 (1%)
Data presented as n (%).* M. hominis*: *Mycoplasma hominis*, *M. genitalium*: *Mycoplasma genitalium*, *U. parvum*: *Ureaplasma parvum*, *U. urealyticum*: *Ureaplasma urealyticum*, *C. trachomatis*: *Chlamydia trachomatis*, Data presented as frequency		

**Table 4:** Comprising semen fluid characteristics in infertile and fertile men


**Semen parameter**	**Groups**		**P-value**
	**Fertile**	**Infertile**	
**Volume (mL)**	3.69 ± 1.88	2.74 ± 1.42	< 0.001
**Progressive sperm motility (%)**	45.81 ± 15.05	16.93 ± 11.50	< 0.001
**Non-progressive sperm motility (%)**	17.11 ± 6.98	13.55 ± 9.88	< 0.001
**Immotile sperm (%)**	37.32 ± 12.55	68.30 ± 18.45	< 0.001
**Total progressive motility (%)**	61.31 ± 9.31	30.64 ± 17.25	< 0.001
**Sperm concentration ( × 10^6^/mL)**	65.55 ± 30.39	51.98 ± 47.37	0.01
**Viability (%)**	73.14 ± 10.07	44.04 ± 18.64	< 0.001
Data are presented as Mean ± SD. Mann*-*Whitney U test, P < 0.05 was considered statistically significant. Reference ranges: Volume > 1.5 ml, Progressive sperm motility: > 32, Total progressive motility > 40, Sperm concentration: > 15 × 10^6^/ml, Viability: > 58 (% live)			

**Table 5:** The risk for infertility in infected specimens with bacterial agents


**Bacteria**	**OR (95% CI)**	**P-value**
Mycoplasma genitalium	8.064 (1.7-37)	< 0.001
Mycoplasma hominis	0.274 (0.090-0.932)	0.02
Ureaplasma **spp.**	1.030 (0.490-2.169)	0.93
Ureaplasma urealyticum	1.170 (0.165-8.299)	0.87
Ureaplasma parvum	1.665 (0.692-4.009)	0.25
Chlamydia trachomatis	16.027 (1.67-153179)	0.01
OR (95% CI): Odd ratio (95% confidence interval). P < 0.05 was considered statistically significant. Logistic regression		

## 4. Discussion 

There are many causes of male infertility, of which 8-35% are related to genital tract infections. Although sexually transmitted organisms, in particular, *C. trachomatis, M. genitalium, M. hominis, U. parvum *and *U. urealyticum* may affect spermatogenesis and subsequent fertility in men, the role of these bacteria as the important causes of male infertility is still a controversial issue (13-15). The majority of women and men who are infected with *C. trachomatis*, genital *Mycoplasma* and genital *Ureaplasma* are not aware of their infection because they are asymptomatic. Therefore, detection of these agents is important (8, 16). This study provides information on the prevalence of the above-noted bacteria, their impact on fertility potential, and their association with quality of sperm among asymptomatic fertile and infertile men attending research and clinical centers of fertility in Kerman (southeast of Iran).

In Iran, the prevalence of *C. trachomatis* in low-risk people without any symptoms varies from 6.4%-10.3% (17). In the present study, the frequency of *C. trachomatis* among the infertile group was 9% higher than previous reports from other countries (2, 6) and closely similar to the result reported from Ahvaz, Iran (18).

The prevalence of urogenital mycoplasmas in men varies between 2%-44.3%. The prevalence of *M. genitalium* in our examined infertile men was almost similar to the previous study from Kerman, Iran (14%) (19), lower than reported in Tehran, Iran (20), and higher than Jordan (3.2%) (2), Kuwait (4.7%) (6), and Tunisia (4.8%) (8).

The high prevalence of *C. trachomatis* and genital mycoplasmas in infertile men compared with fertile men has been reported in several studies (2, 9, 21). Our research demonstrated a higher detection rate for *C. trachomatis* and *M. genitalium* in semen samples of infertile men than the control group. Similar to previous findings in Iran and the other countries (2, 18, 20-21), a significant difference was observed in the frequency of these microorganisms in the infertile and fertile groups (p < 0.001). However, these results are inconsistent with some studies that did not report any difference between these two groups (6, 22). These differences can be due to the diversity of the participants and type of samples, using techniques with different sensitivity and specificity, the cultural and geographical features of the countries, and multiple sexual partners (1, 18).

Our study demonstrated that the prevalence of *Ureaplasma* spp. in infertile men is high. Herein, we differentiate different *Ureaplasmas* spp. in infertile men, while most of the previous studies did not discriminate between *U. urealyticum* and *U. parvum* (9, 13). Our study indicated that *U. parvum* species was more common than *U. urealyticum* which agrees with the previously reported studies (2, 9) and is in contrast to other studies that found *U. urealyticum* species to be more common (8, 10). In our study, no statistically significant difference was found between infertile and fertile men in the prevalence of *M. hominis* and *U. urealyticum* (7% vs. 16% and 2% vs. 3%, respectively). Our data are in accordance with previously reported studies (9, 23), while they are not in agreement with the study by Moosavian and co-workers (18).

The association between *M. genitalium* and male infertility has been investigated in a few studies, but, unlike our study, these studies did not have any control group (8, 24). Our data showed that the risk for infertility in infected men with *M. genitalium* was 8.06 times higher than in non-infected men (OR = 8.06, CI: 37-1.7). The effect of *M. genitalium* on the obstructive inflammatory process is far greater than its effect on functional parameters of semen (8). Although *C. trachomatis* attaches to the surface and the nucleolus of spermatozoa, its role in infertility is unclear (25). In our study, logistic regression analysis showed that the risk of infertility in infected men with *C. trachomatis* was 16 times higher than in non-infected men*.*

Furthermore, we investigated the impact of infection on sperm quality. There are conflicting results in this field. Some studies have revealed no relationship between the presence of these bacteria and semen quality (8, 23). Nevertheless, others have proven the effect of these bacteria on semen parameters (21, 26). Our study was consistent with the latter groups, as it was shown that *M. genitalium* and *M. hominis* were associated with reducing progressive sperm motility and semen volume, respectively.

Also, similar to several studies, *C. trachomatis* was associated with reduced progressive sperm motility (1, 18, 27). In a study by Liu and co-workers, the comparison between *C. trachomatis* infected and non-infected specimens revealed that the prevalence of *C. trachomatis* infection was related to the reduction in sperm vitality, but the difference was not statistically significant (23). However, in our study, *C. trachomatis* was associated with reduced sperm vitality.

Mycoplasmas in urogenital tracts of infertile men may negatively affect semen volume, pH, sperm motility, morphology, concentration, and vitality (28). In this study, semen volume in infertile men infected with *M. hominis* was significantly lower compared to non-infected infertile men.

One of the important agents in the natural fertilization cycles is progressive sperm motility. Similar to one study (9), *Ureaplasma *species had the same effect on semen quality except for *U. parvum *infection, which correlated with abnormal sperm morphology. The capability of attachment of bacteria to spermatozoa is different, and their influence on some of the seminal fluid parameters, host factors, and cellular interactions varies (18).

As the bacterial load may affect the outcome of the infection (9, 29), lack of knowledge about the bacterial load in infected specimens is a limitation in our study. Quantitative assessments either by quantitative culture or real-time PCR are recommended.

## 5. Conclusion

According to our results, *C. trachomatis*, genital mycoplasmas, and genital ureaplasmas are widely present in infertile men in Kerman, Iran. Because of the significant destructive effects of bacteriospermia on males' reproductive health, informing people about urogenital infections and protection ways, screening and treating asymptomatic cases seem to be indispensable. According to our results, the clinician should consider *C. trachomatis* and *M. genitalium* in men with decreased sperm progressive motility and viability during the infertility assessment.

##  Conflict of Interest 

The authors declare that there is no conflict of interest.
